# Nutrition habits in 24-hour mountain bike racers

**DOI:** 10.1186/2193-1801-3-715

**Published:** 2014-12-09

**Authors:** Daniela Chlíbková, Beat Knechtle, Thomas Rosemann, Ivana Tomášková, Vlastimil Chadim, Marcus Shortall

**Affiliations:** Centre of Sports Activities, Brno University of Technology, Brno, Czech Republic; Institute of Primary Care, University of Zurich, Zurich, Switzerland; Gesundheitszentrum St. Gallen, St. Gallen, Switzerland; Faculty of Forestry and Wood Sciences, Czech University of Life Sciences, Prague, Czech Republic; Department of Preventive Medicine, Faculty of Medicine, Masaryk University, Brno, Czech Republic; Institute of Technology Tallaght, Dublin, Ireland; Facharzt FMH für Allgemeinmedizin, Gesundheitszentrum St. Gallen, Vadianstrasse 26, 9001 St. Gallen, Switzerland

**Keywords:** Race nutrition, 24-hour race, Ultra-cycling

## Abstract

We investigated seventy-four ultra-mountain bikers (MTBers) competing in the solo category in the first descriptive field study to detail nutrition habits and the most common food before during and after the 24 hour race using questionnaires. During the race, bananas (86.5%), energy bars (50.0%), apples (43.2%) and cheese (43.2%) were the most commonly consumed food, followed by bread (44.6%), rice (33.8%) and bananas (33.8%) after the race. Average fluid intake was 0.5 ± 0.2 l/h. The main beverage was isotonic sports drink (82.4%) during and pure water (66.2%) after the race. The most preferred four supplements in the four weeks before, the day before, during and after the race were vitamin C (35.1%), magnesium (44.6%), magnesium (43.2%) and branched-chain amino acids (24.3%), respectively. Total frequency of food intake (30.6 ± 10.5 times/24 hrs) was associated with fluid intake (*r* = 0.43, *P* = 0.04) and both were highest at the beginning of the race and lower during the night hours and the last race segment in a subgroup of twenty-three ultra-MTBers. Supplement intake frequency (6.8 ± 8.4 times/24 hrs) was highest during the night hours and lower at the beginning and end of the race. Elevated food and fluid intake among participants tracked across all race segments (*P* < 0.001). In conclusion, the nutrition strategy employed by ultra-MTBers was similar to those demonstrated in previous studies of ultra-cyclists with some exceptions among selected individuals.

**Electronic supplementary material:**

The online version of this article (doi:10.1186/2193-1801-3-715) contains supplementary material, which is available to authorized users.

## Background

The continuous physical stress in ultra-endurance performances leads to an unavoidable energy deficit (Knechtle 2013). In most ultra-endurance exercises, energy intake is lower than energy expenditure (Bescós et al. 2012[a], [b]; Bircher et al. 2006; Enqvist et al. 2010; Francescato et al. 2002; Linderman et al. 2004). Ultra-endurance sports such as 24-hour bike races require large caloric intakes in order to match the expenditures required to complete these prolonged events (Bescós et al. 2012[a], [b]; Bircher et al. 2006; Francescato et al. 2002; Linderman et al. 2004; White et al. 1984). The type, timing and amount of foods ingested as well as the co-ingestion of ergogenic aids such as caffeine are important factors directly linked to sport performance in endurance events (Eberle 2007; Jeukendrup 2014; Laursen et al. 2001; Maughan et al. 2011; Ormsbee et al. 2014; Peters 2003). At present, comprehensive dietary macronutrient recommendations for ultra-endurance and adventure races are missing. As a result, race recommendations for carbohydrate (Burke 2002; Burke et al. 2011; Jeukendrup 2004, 2011, 2014; Lambert et al. 2003; Millard-Stafford et al. 2008; Peters 2003), protein (Ormsbee et al. 2014; Ranchordas 2012) and fat (Ormsbee et al. 2014; Lambert 2003) intake have been extrapolated from studies on traditional endurance events.

The nutritional strategy during a 24-hour race and a defined plan for feeding can be a key strategy to finish a race successfully (Burke 2002; Eberle 2007; Jeukendrup 2011). On the contrary, it is necessary to be flexible and open to changes if things are not working according to a defined plan. Many ultra-mountain bikers (ultra-MTBers) exhibit an over-reliance on sports supplements compared to traditional foodstuffs and/or they can expect to face extreme nutrient demands to achieve their fuel and fluid replacement goals. In events lasting 8–24 hrs it is not necessary and practical to meet total energy expenditure (Burke 2002). The main goal is to match muscle fuel needs for carbohydrate (Burke 2002; Eberle 2007; Jeukendrup 2011; Stuempfle et al. 2011); however, there are many practical hurdles which must be overcome in order to achieve this goal (Burke 2002; Eberle 2007). In events such as triathlons or road cycling races it is easier to eat solid food while cycling. However, in a mountain bike race, the course is too technical to allow ample opportunity for feeding (Burke 2002). Competitors are more reliant on feed stations or food provided by their support crew or themselves at pit stops. Factors such as appetite suppression and gastrointestinal distress can also reduce the dietary intake during longer competition (Bescós et al. 2012a; Eberle 2007; Jeukendrup 2011, 2014).

Information about energy intake in ultra-endurance cyclists during a 24-hour race is scarce (Bescós et al. 2012a, [b]; Burke 2002; Knechtle et al. 2003; White et al. 1984). Most research conducted on nutrition during extreme endurance is based on case studies and studies involving a small number of individuals (Bescós et al. 2012a, [b]; Rehrer 2001; White et al. 1984). In relation to energy demands, several studies have assessed the nutritional requirements and behavior of cyclists during solo events (Francescato et al. 2002; Havemann et al. 2008; Knechtle et al. 2005; 2007; White et al. 1984). However, data on race diet in a solo category in a 24-hour mountain bike races are lacking. Therefore, the aim of this study was to investigate dietary, fluid and ergogenic aid intakes (1) one month prior, (2) one day prior, (3) during and (4) during the three hours following the race. We hypothesized that dietary intake and the use of supplements would not differ to current nutritional recommendations for long-distance events. Additionally, we evaluated the amount of fluid ingested, and the frequency of ingestion of solid food and supplements in four race segments during 24 hours. We predicted a lower fluid intake and a frequency of food intake during the night and a higher frequency of supplement intake in the last race segment before the end of the race.

## Methods

Research within the project proceeded in an accordance with the law (No. 96/2001 Coll. M. S. on Human Rights and Biomedicine and Act No. 101/2000 Coll. Privacy) and the study was approved by the local institutional ethics committee (The Ethics Committee of the Faculty of Masaryk University, Brno, Czech Republic). Ultra-MTBers were notified approximately three months before the race of the study via an e-mail and were informed about the planned investigation with indication that participation is voluntary. Prior to the race, athletes were informed of the procedures and gave their informed written consent.

In the current study, we observed the nutritional habits of entrants in two 24-hour MTB races held in the Czech Republic in 2012. The first measurement was performed at the ‘Czech Championship 24-hour MTB’ in Jihlava (Race 1). The ultra-MTBers started on May 19th 2012 at 12:00 and finished on May 20th 2012 at 12:00. The course was primarily held on a 9.5 km single-track trail with an elevation of 220 m. The second measurement was performed at the 24-hour race ‘Bike Race Marathon MTB’ in Liberec (Race 2) taking place from June 9th 2012 to June 10th 2012. The course was comprised of a 12.6 km loop with an elevation of 250 m. Nearby temperature during both races ranged from 6°C to 23°C. We acknowledge that the use of the temperatures from the nearest local weather stations underestimated the actual temperatures through some parts of the course. A single aid station was positioned at the start/finish area, where a variety of food and beverages such as hypotonic sports drinks, tea, soup, caffeinated drinks, water, fruit, vegetables, energy bars, bread, soup, sausages, cheese, bread, chocolate and biscuits were available. The ultra-MTBers could also use their own supplies during pre-determined breaks. In total, 77 racers consented to participate in the present study (*i.e*. 40 in Race 1 and 37 in Race 2).

Volunteers were asked to complete a training diary in the three-month period before the race. The training records consisted of sex, age, the number of years that they had actively participated in cycling, their best result (measured in km) in a 24-hour race, the number of hours spent by training per week as well as the number of hours of cycling training per week. The MTBers also completed a questionnaire about their pre-race diet and the use of supplements before the race. Prior to the start of the race, a second questionnaire about race and post-race nutrition was administered to the participants by trained personnel. The MTBers and their support teams marked items and noted the amount of fluid and the frequency of food and supplements consumed at the aid station or provided by the support crew. For the purpose of recording, the race was broken into discrete segments (*i.e*. from start at 12.00 hrs to 18.00 hrs, from 18.00 hrs to 24.00 hrs, from 24.00 hrs to 6.00 hrs and from 6.00 hrs to 12.00 hrs the next day). The subjects were not told what food or fluid to consume during the race. During the event, the time and race speed taken by each athlete to complete each lap were recorded by a personal lap counter (Sportsoft timing spol. s.r.o. chip technology). After the race, the ultra-MTBers sent their completed questionnaires and reports by fax, postal letter or e-mail to the investigator. Participants were instructed to respond within ten days of the event.

### Statistical analyses

The statistical software Statistica (StatSoft, Inc., Tulsa, OK USA) was used for the statistical analysis. Differences between pre-race variables (sex, age, years as an active biker, best result in a 24-hour race, average weekly training hours and average weekly cycle hours) in the racers from the two races in the subgroup (*n* = 23) were calculated using one-way ANOVA after fulfilling two basic presumptions – homogeneity of variance (Levene test) and the normality of distribution (Shapiro-Wilks test). The association between pre-race characteristics and the amount of fluid intake, the frequency of food or supplement intake was calculated using Spearmans rank correlation coefficient. Spearman correlation coefficient was used to reveal the measure of statistical dependence between race speed and fluid intake, between the frequency of food and/or supplements intake and the amount of fluid intake mutually, between total nutrition intake and intake in all race segments. Results in the text are presented as mean ± SD. For all statistical tests, significance was set at a level of 0.05.

## Results

Seventy-four (96.1%) of the 77 race participants successfully finished the race and returned their completed questionnaires. One MTBer was forced to retire due to an equipment failure, while two had medical complications. Fluid intake and the frequency of food and supplement intake were evaluated in a subgroup of 23 (13 males and 10 females) ultra-MTBers (30.1%). Incomplete questionnaires were excluded from the final analysis. We found no significant differences in pre-race variables (*i.e*. sex, age, years as an active biker, best result in a 24-hour race, average weekly training hours and average weekly hours spent cycling per week) between the racers from two races in this subgroup (*P* > 0.05). Pre-race characteristics and an average race speed are presented in Table 1.

**Table 1:** **The pre-race and race characteristics of the soubgroup (** ***n*** **= 23)**

Parameter	Unit	All ultra-MTBers (***n*** = 23)	Male ultra-MTBers (***n*** = 13)	Female ultra-MTBers (***n*** = 10)
Age	yrs	36.3 ± 6.6	35.4 ± 6.5	37.3 ± 6.6
Years as an active biker	yrs	7.6 ± 2.9	8.0 ± 3.0	7.2 ± 2.7
Personal best average km in a 24-hour race	km	280.8 ± 87.2	313.6 ± 82.9	238.1 ± 73.1
Training hours weekly	h	11.3 ± 3.6	10.6 ± 4.3	12.3 ± 2.2
Cycling hours weekly	h	10.0 ± 3.2	9.7 ± 3.8	10.5 ± 2.0
Race speed	km	15.8 ± 1.7	16.5 ± 1.7	15.3 ± 1.3

*Note*: data are presented as mean ± standard deviation.

### Pre-race dietary intake and supplementation (*n* = 74)

In the four weeks preceding the race, only two ultra-MTBers (2.7%) followed no special pre-race diet (Table 2). Forty-seven racers (63.5%) used a carbohydrate-rich diet and twenty-five athletes (34.8%) accomplished a protein- and (or) fat-rich diet. Forty-five (60.8%) racers consumed ergogenic supplements with a preference for branched-chain amino acids (BCAA) (21.6%) and L-Carnitine (12.2%). Fifty-one (68.9%) racers ingested vitamins and favoured vitamin C (35.1%), multi-vitamin products (18.9%) and all kinds of vitamin B (14.9%). Fifty (67.6%) ultra-MTBers consumed minerals with a special preference for magnesium (32.4%), multi-mineral products (13.5%) and potassium (10.8%) (Table 3).

**Table 2:** **Diet in four weeks and the day before the start of race (** ***n*** **= 74)**

Kind of diet	Four weeks before the race	Percentage (%)	The day before the race	Percentage (%)
Carbohydrate-rich diet	47	63.5	62	83.8
Protein-and (or) fat-rich diet	25	33.8	12	16.2
No special diet	2	2.7	0	0

**Table 3:** **Supplementation before, during and after the race (** ***n*** **= 74)**

Kind of supplementation	Four weeks before the race	Percentage (%)	The day before the race	Percentage (%)	During the race	Percentage (%)	After the race	Percentage (%)
**Ergogenic supplements**								
Concentrate of AA	5	6.8	3	4.1	5	6.8	5	6.8
BCAA	16	21.6	13	17.6	22	29.7	18	24.3
Coenzym Q10	5	6.8	4	5.4	1	1.4	2	2.7
L-Carnitine	9	12.2	8	10.8	15	20.3	4	5.4
Glutamine	4	5.4	2	2.7	2	2.7	1	1.4
Concentrate of EFA	5	6.8	2	2.7	2	2.7	1	1.4
Caffeine tablets	0	0	0	0	2	2.7	0	0
Lecithin	0	0	0	0	1	1.4	0	0
Glucose	0	0	0	0	3	4.1	0	0
Creatine	4	5.4	3	4.1	5	6.8	1	1.4
*No intake of ES*	29	39.2	49	66.2	18	24.3	46	62.2
**Vitamins**								
Multi-vitamin	14	18.9	9	12.2	8	10.8	8	10.8
Vitamin C	26	35.1	23	31.1	9	12.2	13	17.6
Vitamin B (complex)	11	14.9	9	12.2	5	6.8	9	12.2
Vitamin E	3	4.1	3	4.1	2	2.7	4	5.4
Folic acid	2	2.7	2	2.7	2	2.7	2	2.7
*No intake of vitamins*	23	31.1	35	47.3	57	77.0	51	68.9
**Minerals**								
Multi-mineral	10	13.5	7	9.5	8	10.8	3	4.1
Magnesium	24	32.4	33	44.6	32	43.2	14	18.9
Calcium	7	9.5	3	4.1	5	6.8	4	5.4
Iron	5	6.8	1	1.4	0	0	1	1.4
Zinc	5	6.8	4	5	3	4.1	1	1.4
Salt tablets	0	0	0	0	6	8.1	1	1.4
Potassium	8	10.8	3	4.1	4	5.4	3	4.1
*No intake of minerals*	24	32.4	25	33.8	18	24.3	60	81.1

*Note*: concentrate of AA – concentrate of amino acid, BCAA – branched-chain amino acid, concentrate of EFA – concentrate of Essentials fatty acid, no intake of ES – no intake of ergogenic supplements.

The day before the race, all ultra-MTBers followed a planned dietary routine, sixty-two (83.8%) racers accomplished a carbohydrate-rich and twelve racers (16.2%) a protein-rich diet. Twenty-five (33.8%) ultra-MTBers consumed ergogenic supplements with a preference for BCAA (17.6%) and L-Carnitine (10.8%). Thirty-nine (52.7%) racers ingested vitamins and favoured vitamin C (31.1%), multi-vitamin products (12.2%) and B vitamins (12.2%). Forty-nine (66.2%) ultra-MTBers consumed minerals with a special preference for magnesium (44.6%) and multi-mineral products (12.2%) (Table 3).

### In-race dietary intake and supplementation (*n* = 74)

**Figure 1:**
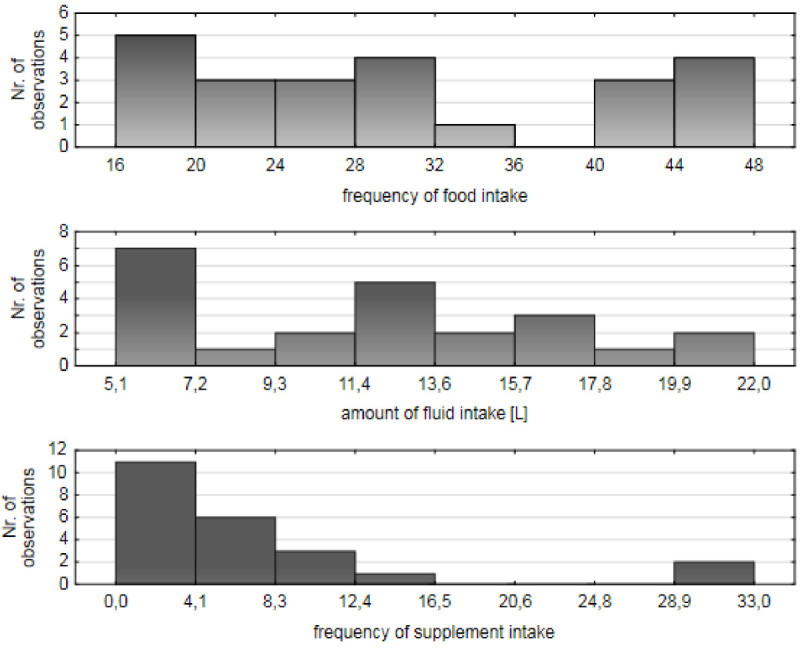
**The frequency of food intake during 24**-**hour mountain bike race. The amount of fluid intake during 24-hour mountain bike race. The frequency of supplement intake during 24-hour mountain bike race.** *Note*: number of testing subjects (*n* = 23).

**Figure 2:**
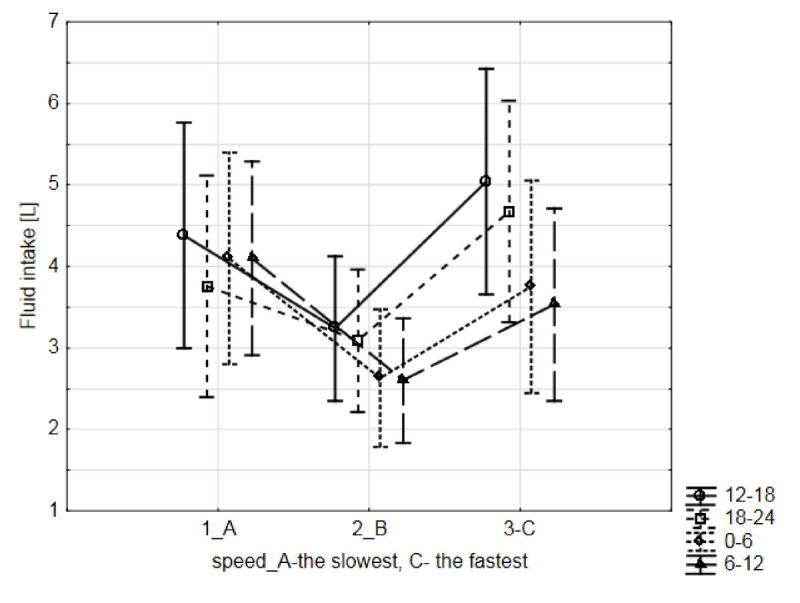
**Fluid intake and race speed.** *Note*: group 1A – MTBers with race speed 12.80 – 14.80 km/h, group 1B – MTBers with race speed 14.90 – 16.90 km/h and group 1C – MTBers with race speed 17.00 – 19.00 km/h. Race segments: 1. 12.00 – 18.00 hrs, 2. 18.00 – 24.00 hrs, 3. 24.00 – 6.00 hours, 4. 6.00 – 12.00 hrs. *Note*: number of testing subjects (*n* = 23).

A variety of fifty-six different solid foods and sixteen beverages were consumed during the race (Tables 4 and 5). The ultra-MTBers have achieved an average fluid intake 0.5 ± 0.2 l/h (12.0 ± 5.0 l/24 hrs). The main beverage during the race was isotonic sports drink (82.4%), followed by pure water (71.6%), Coca Cola^®^ (54.1%), and tea (51.4%) (Table 5). The highest intake was 22.0 l/24 hrs and the lowest intake was 5.1 l/24 hrs (Figure 1). The highest intake we recorded during the first segment of the race [S1 (*i.e*. 12.00 -18.00 hrs) 3.5 ± 1.5 l, respectively 0.6 ± 0.2 l/h] with an average fluid consumption decreasing during each subsequent segment [S2 (*i.e*. 18.00 - 24.00 hrs) 3.1 ± 1.4 l equal to 0.5 ± 0.3 l/h, S3 (*i.e*. 24.00 - 6.00 hrs) 2.7 ± 1.3 l, respectively, 0.5 ± 0.3 l/h, S4 (*i.e*. 6.00 - 12.00 hrs) 2.6 ± 1.2 l equal to 0.4 ± 0.2 l/h. Racers were assigned to the three groups based on their race speed (group 1A: MTBers with a race speed of 12.8 - 14.8 km/h, group 1B: 14.9 - 16.9 km/h and group 1C: 17.0 - 19.0 km/h) (Figure 2). Average fluid intake in group 1A was 0.6 ± 0.1 l/h, in group 1B 0.4 ± 0.1 l/h and in group 1C 0.6 ± 0.1 l/h. We found no association between race speed and the amount of fluid intake in the present 24-hour ultra-MTBers (*P* > 0.05) (Figure 2).

**Table 4:** **Food intake during and after the race (** ***n*** **= 74)**

Kind of food	During the race	Percentage (%)	After the race	Percentage (%)
*Fruits and nuts*				
Bananas	64	86.5	25	33.8
Apples	32	43.2	11	14.9
Oranges	18	24.3	8	10.8
Raisins	15	20.3	1	1.4
Grapes	8	10.8	1	1.4
Peaches	2	2.7	1	1.4
Blueberries	1	1.4	0	0
Apricots	2	2.7	0	0
Peanuts	1	1.4	0	0
Pistachio nuts	1	1.4	0	0
Sunflower seeds	1	1.4	0	0
Pears	1	1.4	0	0
Dried fruits	11	14.9	2	2.7
Figs	1	1.4	0	0
Strawberries	1	1.4	0	0
Pineapple	18	24.3	5	6.8
Cherries	0	0	1	1.4
Walnuts	1	1.4	2	2.7
Melon	17	22.9	3	4.1
Kiwi	7	9.5	0	0
Fruit purée	1	1.4	0	0
Grapefruit	2	2.7	1	1.4
*Meat and fish*				
Chicken	14	18.9	25	33.8
Fish	1	1.4	6	8.1
Beef	1	1.4	12	16.2
Pork	7	9.5	14	18.9
Sausages	3	4.1	7	9.5
White pudding	0	0	1	1.4
*Vegetables and salads*				
Potatoes	6	8.1	13	17.6
Beans	0	0	6	8.1
Carrots	5	6.8	5	6.8
Corn	3	4.1	7	9.5
Cucumbers	6	8.1	9	12.2
Tomatoes	13	17.6	12	16.2
Olives	1	1.4	1	1.4
Soy	0	0	3	4.1
Vegetables sushi	1	1.4	0	0
Zucchini	1	1.4	0	0
Oyster mushroom	1	1.4	0	0
Pickles	1	1.4	1	1.4
*Carbohydrate*-*rich food*				
Bread	28	37.8	33	44.6
Noodles	19	25.7	23	31.1
Rice	21	28.4	25	33.8
Carbohydrate gel	10	13.5	0	0
Cake	2	2.7	2	2.7
Biscuits	30	40.5	11	14.9
Pizza	0	0	1	1.4
Chips	2	2.7	6	8.1
Cornflakes	1	1.4	1	1.4
Porridge	7	9.5	5	6.8
Energy bars	37	50.0	10	13.5
Muesli bars	10	13.5	0	0
Soy bars	2	2.7	0	0
Pancakes	2	2.7	0	0
Honey	2	2.7	0	0
Treacle	1	1.4	0	0
*Dairy produce*				
Cheese	32	43.2	18	24.3
Eggs	1	1.4	5	6.8
Yoghurt	0	0	8	10.8
Ice team	0	0	5	6.8
Chocolate	19	25.7	10	13.5
Cottage cheese	1	1.4	0	0
Pudding	1	1.4	0	0
Sour team	0	0	1	1.4

**Table 5:** **Beverages during and after the race (** ***n*** **= 74)**

Beverage	During the race	Percentage (%)	After the race	Percentage (%)
Water	53	71.6	49	66.2
Isotonic sports drink	61	82.4	20	27.0
Coffee	17	23.0	13	17.6
Apple juice	5	6.8	3	4.1
Coca Cola®	40	54.1	27	36.5
Beer	10	13.5	29	39.2
Tea	38	51.4	19	25.7
Soup	34	45.9	22	29.7
Orange juice	1	1.4	4	5.4
Lemonade	2	2.7	5	6.8
Ice tea	1	1.4	2	2.7
Milk	1	1.4	4	5.4
Chocolate milk	1	1.4	2	2.7
Cocktail	1	1.4	2	2.7
Fruit juice (other)	4	5.4	4	5.4
Tomato juice	0	0	1	1.4
Red Bull	20	27.0	4	5.4
Regener drink	0	0	5	6.8

Ultra-MTBers ate with an average frequency 30.6 ± 10.5 times/24 hrs, equally to 1.3 ± 0.4 times/h. Bananas (86.5%) were preferred before energy bars (50.0%), apples (43.2%), cheese (43.2%), biscuits (40.5%) and bread (37.8%) (Table 4). The racer with the highest frequency ate 48 times and the racer with the lowest frequency ate 16 times in the 24 hours (Figure 1). Racers ate the most (9.3 ± 4.9 times) during the first race segment (*i.e*. 12.00 - 18.00 hrs), equal to 1.6 ± 0.8 times/h; with a decrease (6.9 ± 3.8 times) during the second segment (*i.e*. 18.00 - 24.00 hrs), 1.1 ± 0.6 times/h, respectively. A moderate increase appeared during the third segment (*i.e*. 24.00 - 6.00 hrs) 7.0 ± 3.5 times, equal to 1.2 ± 0.6 times/h and during the last fourth segment (*i.e*. 6.00 - 12.00 hrs) 7.3 ± 3.6 times, equal to 1.2 ± 0.6 times/h, respectively.

BCAA were the preferred ergogenic supplement during the race for twenty-two athletes (29.7%), and L-Carnitine for fifteen racers (20.3%). Thirty-two (43.2%) racers consumed magnesium, nine (12.2%) consumed vitamin C, five (6.8%) B vitamins, and eight (10.8%) multi-mineral products (Table 3). The ultra-MTBers supplemented 6.8 ± 8.4 times/24 hrs (Figure 1). The highest achieved frequency by one racer was 33 ± 10.2 times/24 hrs, the lowest intake of supplements was zero times/24 hrs. Supplements were consumed 1.4 ± 2.2 times during the first segment (*i.e*. 12.00 - 18.00 hrs), 2.0 ± 2.3 times during the second segment (*i.e*. 18.00 - 24.00 hrs), 1.9 ± 2.6 times during the third segment (*i.e*. 24.00 - 6.00 hrs) and 1.7 ± 2.2 times during the final race segment (*i.e*. 6.00 - 12.00 hrs).

### Post-race dietary intake and supplementation (*n* = 74)

Forty-three different solid foods and eighteen different beverages were consumed after the race (Tables 4 and 5). The preferred food was bread (44.6%), followed by rice (33.8%), bananas (33.8%), chicken (33.8%), noodles (31.1%), cheese (24.3%), pork (18.9%), and potatoes (17.6%). The main beverage was pure water (66.2%), followed by beer (39.2%), Coca Cola® (36.5%) and soup (29.7%). BCAA were the most preferred ergogenic supplement (24.3%); vitamin C (17.6%) and magnesium (18.9%) were preferably consumed as vitamins and minerals after the race (Table 3).

The total amount of fluid ingested was related to fluid intake in each race segment (S1: *r* = 0.88, *P* < 0.001; S2: *r* = 0.91, *P* < 0.001; S3: *r* = 0.92, *P* < 0.001; S4: *r* = 0.76, *P* < 0.001). Also the total supplement frequency (S1: *r* = 0.91, *P* < 0.001; S2: *r* = 0.84, *P* < 0.001; S3: *r* = 0.93, *P* < 0.001; S4: *r* = 0.89, *P* < 0.001) and the food intake frequency (S1: *r* = 0.61, *P* < 0.001; S2: *r* = 0.79, *P* < 0.001; S3: *r* = 0.74, *P* < 0.001; S4: *r* = 0.49, *P* < 0.001) associated with frequencies in all race segments. Fluid intake was related to the frequency of food intake (r = 0.48, *P* < 0.05), but not related to the frequency of supplement intake (*P* > 0.05). Food intake did not associate with the frequency of supplement intake (*P* > 0.05). We found no significant differences between pre-race variables (sex, age, years as an active biker, best result in a 24-hour race, average weekly training hours and average weekly cycle hours) and the amount of fluid intake, the frequency of food intake, or the frequency of supplement intake (*P* > 0.05). The highest correlation appeared between the amount of fluid intake and the participants best result in a 24-hour race (*r* = 0.24), however, the correlation was not significant (*P* > 0.05).

## Discussion

Pre-race dietary intake did not differ from current nutritional recommendations for long-distance events. However, not all present ultra-MTBers followed a strict carbohydrate-rich diet before the race. Nevertheless, most of the athletes in the current study tried to achieve high concentrations in muscle glycogen prior to the start of the race by the traditional supercompensation protocol, while, a portion of the participants followed protein- and fat-rich diets. Having elevated muscle glycogen stores at the start may be beneficial for endurance performance, although this does not necessarily have to be achieved by the traditional supercompensation diet (Jeukendrup 2011). Well-trained endurance athletes can achieve a glycogen supercompensation without the need for the depletion phase prior to loading (Burke et al. 2011; Kreider 1991) and a higher carbohydrate intake might not always result in a better performance (Jeukendrup 2011). In a study by Knechtle et al. (2007), fifty percent of the successful finishers did not follow a carbohydrate-rich diet prior to competing in the‘ Race across America‘ (RAAM).

No consistent performance benefit has been shown following a high-fat diet prior to an endurance performance (Ormsbee et al. 2014; Peters 2003). Interventions involving increased fat availability have failed to demonstrate ergogenic benefits in performance despite reduced carbohydrate utilization during exercise, increased free fatty acid levels in the blood and an increased lipid metabolism during exercise (Hargreaves et al. 2004; Lambert et al. 1994; Phinney et al. 1983). Dietary fat intake over a 24-hour period increased muscle triglyceride stores, but reduced cycling time-trial performance in seven endurance-trained men completing a 120-min cycling bout, compared with a high carbohydrate diet (Starling et al. 1997). The effects on subsequent exercise performance are equivocal (Ormsbee et al. 2014) and may show benefits for moderate-intensity in an ultra-distance setting, which favors fat as the primary fuel source (Hawley et al. 1995). Carbohydrate and protein ingestion prior to exercise could potentially enhance exercise performance in humans by augmenting and/or sparing glycogen stores (Rowlands and Hopkins 2002). Conversely, despite substantial effects on plasma hormone concentrations and fuel utilization, high-fat, high-protein and high-carbohydrate pre-exercise meals have no clear effect on cycling performance (Rowland et al. 2002). Nevertheless, current evidence continues to support mandatory high carbohydrate intakes before an event to maximize muscle and liver glycogen stores (Peters 2003). Carbohydrate intakes prior to endurance exercise have generally been shown to enhance performance, despite increasing insulin levels and reducing fat oxidation (Ormsbee et al. 2014).

Fluid and food intake was similar to common nutrition habits of ultra-cyclists with some exceptions in the current study. Dietary intake was highest at the beginning of the race and lower during night hours, in the middle of the race (the second and the third race segments) and at the end of the race (the last race segment). In the ultra-MTBers with the highest dietary intake, this characteristic tracked across all four segments. Average fluid intake was in an accordance with the International Marathon medical Directors Association (Hew-Butler et al. 2006). The present ultra-MTBers preferred carbohydrate calories originating from fruits (*e.g*. bananas, apples, oranges and raisins), from energy beverages (*e.g*. isotonic sports drinks and Coca Cola^®^), from energy bars, carbohydrate gels, or from other carbohydrate-rich foods such as biscuits and bread. The most frequently consumed food in the present ultra-MTBers were bananas, which was in an agreement with Knechtle et al. study about the ultra-cycling race‘ RAAM‘ (Knechtle et al. 2007). Cheese, a food that contains more dietary fat than carbohydrate, was the third most ingested food during the present 24-hour race.

After the race, the most consumed food was bread, followed by chicken, with apples being the third preferred food in the present study. Carbohydrates are considered to be the most important fuel during ultra-endurance exercise and it is essential to ingest carbohydrate (Applegate 1991; Burke et al. 2001; Cermak and van Loon 2013; Jeukendrup 2010, 2011, 2014; Kreider 1991; Pfeiffer et al. 2012; Stellingwerff and Cox 2014). Nevertheless, cheese was also mentioned as a favorite food during the race and chicken was preferred even before bread in contrast to the present study in the ultra-endurance road cyclists competing in the‘ RAAM‘ (Knechtle et al. 2007). The energy demands of a 24-hour cycling event under laboratory conditions (White et al. 1984) represented near maximal levels of sustainable ergogenic effort by a cyclist in a case study. Most of the carbohydrate intake during an Ironman triathlon occurred during the cycling leg, where intake was almost three times as high as during the running leg (Kimber et al. 2002). Pfeiffer et al. (2012) demonstrated that higher carbohydrate intakes occur in cycling and triathlon events compared marathons and that better performance in Ironman races correlated with greater carbohydrate intake. With carbohydrate feeding during cycling, it has repeatedly been shown that muscle glycogen breakdown is unaffected (Jeukendrup 2011). Performance nutrition is based on evidence that increased carbohydrate availability enhances endurance and performance (Burke et al. 2001; Cermak and van Loon 2013; Jeukendrup 2014; Pfeiffer et al. 2012). The availability of carbohydrate as a substrate for muscle and the central nervous system is an important factor in endurance performances enduring for longer than 90 min (Burke et al. 2001). On the contrary, the role of protein metabolism during ultra-endurance race is not clear (Kreider 1991; Rowland et al. 2002). Endurance exercise results in the oxidation of several amino acids; however, the total amount of amino acid oxidation during endurance performance amounts to only 1-6% of the total energy cost of exercise (Tarnopolski 2004). A higher protein consumption can be associated with a reduction of food intake and an increase of the risk of gastrointestinal disturbances (Jeukendrup 2011, 2014; Pfeiffer et al. 2012). In the study of Saunders et al. (2004), a carbohydrate beverage with additional protein calories produced significant improvements in fifteen male cyclists riding on a cycle ergometer in time to fatigue and reductions in muscle damage. However, it was not clear whether these effects were the result of the higher total calorie content of the beverage or due to specific protein-mediated mechanisms. Some, but not all (Breen et al. 2010), studies investigating the effects of post-exercise carbohydrate-protein intake on exercise performance have also noted an enhanced performance (Berardi et al. 2008), possibly as a result of an increased glycogen resynthesis (Berardi et al. 2006). Most of the present ultra-MTBers commercial carbohydrate drinks like the cyclists in the‘ RAAM‘ (Clark et al. 1992), but not in study by Knechtle et al. (2007), where they preferred pure water. The main beverage differed during the race, the ultra-MTBers preferred isotonic sports drinks, and after the race, the athletes preferred pure water. In a study perfomed by Clark et al. (1992), the road cyclists also preferred concentrates of carbohydrates such as carbohydrate drinks and sports bars during the race. The use of multiple transportable carbohydrates is beneficial in prolonged exercise, nevertheless with individual exceptions (Stellingwerff and Cox 2014).

Our hypothesis that fluid intake would be lower during the night and the last segment of the race was confirmed. On the contrary, we did not found a relationship between fluid intake and race speed in present ultra-MTBers. The frequency of solid food was similarly highest during the first segment of the race. The lowest frequency of fluid intake was at the end of the race, food intake frequency was lowest during the second segment of the race; however, with only moderate differences in the last two segments. The decrease in food and fluid intake in some race segments in this study may have been attributable to fatigue, or gastrointestinal distress (Burke et al. 2011). This theory can be supported by the positive association between the fluid amount and the frequency of food intake. These results suggest that ultra-MTBers who had no problems ingesting food were also more likely to drink more and conversely, those with nausea, problems with food intake, lack of support team were more likely to have not eaten and drank less. Notwithstanding, racers adhered to their diet plan during all race segments, with decreasing amounts of food and drink being ingested during night hours and towards the end of the race. Other reasons for decreased dietary intake could also have been lack of familiarity with feeding at night and also altered biological rhythms associated with being awake for more than twenty four hours. Supplement intake was highest in the second and the third race segments, during the night hours, in the middle of the race, which was the inverse of food and fluid intake. We hypothesized the possibility of the replacement of solid food and fluid by ergogenic supplements in the present ultra-MTBers. However, we found no significant associations between the supplement frequency and the amount of fluid intake or the frequency of food intake. We presumed that the higher frequency of supplement intake in the last race segment before the end of the race could be attributed to possible nausea and problems associated with eating at that time. The hypothesis was not confirmed, all intakes (fluid, food and supplement) were lower during the last race segment and racers were presumably too tired to keep any dietary guidelines. We observed large differences in the amount of fluid ingested as well as food and supplement frequency among the individual ultra-MTBers. For example, food frequency was three times higher and supplement intake was even thirty-three times higher in some cases than in other ones during the race. On the contrary, an interesting finding was the fact that thirty-nine percent of the racers in the four weeks before the race, sixty-six percent the day before, twenty-four percent during and sixty-two percent after the race did not use any ergogenic supplements. Finally, some competitors entered the race without a prepared fuelling protocol and according to Burke (2002) next reason could be various race nutrition plan or MTBers without any diet plan. Athletes who are racing to win have different nutrition strategies compared to those who are competing to finish the race (Burke 2002).

The most preferred supplements prior, during and after the 24-hour MTB race were vitamin C, magnesium and branched-chain amino acids. Intake of supplements such as vitamin C, multi-vitamin, vitamin B (complex) and minerals (*e.g*. magnesium and multi-mineral products) is widespread in athletes (Frohnauer et al. 2008; Singh et al. 1993). In a study investigating Triple Iron ultra-triathletes, there was no association between race performance and vitamin and mineral ingestion in the four weeks leading up to the race. In studies by Frohnauer et al. (2008) and Knechtle et al. (2008), ultra-runners with a regular intake of vitamin and mineral supplements in the four weeks before the race finished the competition no faster than athletes without intake of vitamins and minerals. Moreover, in recent studies vitamin C supplementation decreased training efficiency in runners because it prevented some cellular adaptations to exercise (Gomez-Cabrera et al. 2008). The results of a study in endurance runners by Paulsen et al. (2014) showed that vitamin C and E supplements blunted elevations in mitochondrial protein content, which is needed for improving muscular endurance. Furthermore, VO_2_max and running performance were not detectably affected by the supplementation, and the authors pointed out that high dosages of vitamin C should be used with caution. The preferred supplement the day before and during the race in the present study was magnesium. Magnesium supplementation, in general, does not enhance sport performance in well-nourished athletes (Nielsen and Lukaski 2006; Williams 2005), however, marginal magnesium deficiency has been shown to impair performance and amplify the negative consequences of strenuous exercise. The most consumed supplement among present ultra-MTBers after the race were branched-chain amino acids. No studies have attempted to identify levels of BCAA intake that might produce adverse effects on the brain (Fernstrom 2005). Several studies reported a reduced increase in myocellular enzymes, plasma creatine kinase (Saunders et al. 2007) and myoglobin (Valentine et al. 2008) in cyclists after the combined ingestion of carbohydrates and protein. The present ultra-MTBers probably chose to ingest BCAA to enhance their performance (Matsumoto et al. 2009; Tarnopolsky 2004), to protect their muscle mass (Shimomura et al. 2004) and to promote synthesis of muscle protein (Shimomura et al. 2004). During renal failure, an oral supplementation of BCAA can improve appetite and nutritional status (Cano et al. 2006). Pre-race BCAA supplementation may increase subsequent competitive performance when ingested for a longer period of time high-intensity resistance training (overreaching) (Sharp et al. 2010). In contrast, Knechtle et al. (2012) concluded that BCAA-supplementation before and during a 100-km ultra-marathon had no effect on performance, skeletal muscle damage or renal function. Although BCAA also have biochemical and functional effects on the brain, few attempts have been made to characterize time-course or dose–response relations for such effects (Fernstrom 2005).

The third most drank beverage during and after the race in the present study was Coca Cola^®^. Little is known about the intake of ergogenic supplements and their effect on ultra-endurance performance (Singh et al. 1993). In addition, only a few supplements have been evidenced to enhance performance in the absence of harm (Maughan et al. 2011). The ergogenic potential of caffeine on endurance performance lasting approximately one hour has been well documented (Burke 2008, Conway et al. 2003, Cox et al. 2002, Desbrow et al. 2012, Ganio et al. 2009, Goldstein et al. 2010; Meeusen et al. 2013; Pink et al. 2010) and recent research on the effects of pre-exercise caffeine consumption generally supported it as an ergogenic aid (Ormsbee et al. 2014). Caffeine does not improve maximal oxygen capacity directly, but could permit the athlete to train longer in activities lasting longer than two hours. Caffeine intake during sport events has become popular and practical, athletes use ordinarily specialized foods like sports drinks and gels with caffeine and carbohydrates and replacing sports drink with Coca-Cola® during the latter stages of exercise is equally effective in enhancing endurance performance (Cox et al. 2002). The performance-enhancing effects of caffeine reside in the brain, although more research is necessary to reveal the exact mechanisms through which the central nervous system effect is established (Meeusen et al. 2013). An intake of caffeine before and also during exercise improves cycling performance (Conway et al. 2003; Cox et al. 2002; Desbrow et al. 2012). The competitive inhibition of adenosine receptors and subsequent central nervous system stimulation may explain the reported suppressed feelings of discomfort and pain experienced (Astorino et al. 2012) and the attenuated ratings of perceived exertion (RPE) (Stadheim et al. 2013) during exercise with pre-exercise caffeine consumption. Caffeine in the form of sugar sweetened carbonated beverages (Coca Cola®, Red Bull) and carbohydrate bars and gels was therefore a commonly used ergogenic supplement during and after the race in the current study.

### Limitations

The limitation of the study is the use of the self-perception of the athletes to define the type of diet followed in the 3 month period prior to the race.

## Conclusions

The dietary intake and use of supplements seen in the present study were similar to common recommendations for ultra-cyclists, with exceptions according to each athlete's individual nutrition habits. The ultra-MTBers who drank more, had a higher frequency of food intake. In all participants, increased levels of food, fluid and supplement ingestion tracked across all segments of the race and *vice versa*. Future studies need to use employ a more accurate method of measuring and recording dietary intake in order to estimate macronutrient, micronutrient, mineral and supplement intake during ultra-endurance races.

## Authors’ original submitted files for images

Below are the links to the authors’ original submitted files for images. Authors’ original file for figure 1 Authors’ original file for figure 2
