# Supplementary figures and images for: Nutrition habits in 24-hour mountain bike racers
Source: Springerplus. 2014 Dec 9;3:715. doi: 10.1186/2193-1801-3-715 (PMC4320206; doi:10.1186/2193-1801-3-715)

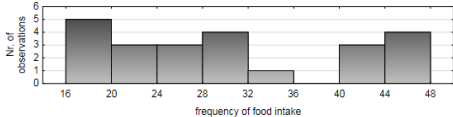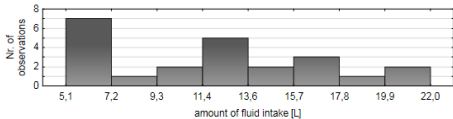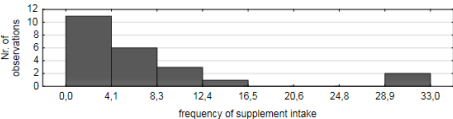

Supplement: Supplementary file 1 — Authors’ original file for figure 1 [file 40064_2014_1483_MOESM1_ESM.pdf]

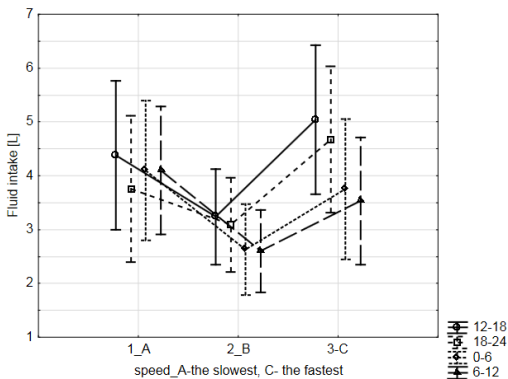

Supplement: Supplementary file 2 — Authors’ original file for figure 2 [file 40064_2014_1483_MOESM2_ESM.pdf]
